# Spatial Dependence of Log-Transformed Electromyography–Force Relation: Model-Based Sensitivity Analysis and Experimental Study of Biceps Brachii

**DOI:** 10.3390/bioengineering10040469

**Published:** 2023-04-12

**Authors:** Chengjun Huang, Maoqi Chen, Zhiyuan Lu, Cliff S. Klein, Ping Zhou

**Affiliations:** 1 Department of Neuroscience, Baylor College of Medicine, Houston, TX 77030, USA; 2 School of Rehabilitation Science and Engineering, University of Health and Rehabilitation Sciences, Qingdao 266072, China; 3 Guangdong Work Injury Rehabilitation Center, Rehabilitation Research Institute, Guangzhou 510440, China

**Keywords:** surface electromyography (EMG), motor unit, spatial distribution, EMG-force relation, biceps brachii

## Abstract

This study investigated electromyography (EMG)–force relations using both simulated and experimental approaches. A motor neuron pool model was first implemented to simulate EMG–force signals, focusing on three different conditions that test the effects of small or large motor units located more or less superficially in the muscle. It was found that the patterns of the EMG–force relations varied significantly across the simulated conditions, quantified by the slope (*b*) of the log-transformed EMG-force relation. *b* was significantly higher for large motor units, which were preferentially located superficially rather than for random depth or deep depth conditions (*p* < 0.001). The log-transformed EMG–force relations in the biceps brachii muscles of nine healthy subjects were examined using a high-density surface EMG. The slope (*b*) distribution of the relation across the electrode array showed a spatial dependence; *b* in the proximal region was significantly larger than the distal region, whereas *b* was not different between the lateral and medial regions. The findings of this study provide evidence that the log-transformed EMG–force relations are sensitive to different motor unit spatial distributions. The slope (*b*) of this relation may prove to be a useful adjunct measure in the investigation of muscle or motor unit changes associated with disease, injury, or aging.

## 1. Introduction

Muscle force is produced by the contraction of muscle fibers belonging to different motor units. Large motor units have higher activation thresholds with faster and larger twitch (Type II) muscle fibers, compared to small motor units that have lower activation thresholds with slower and smaller twitch (Type I) muscle fibers [1,2]. The distribution of large and small motor units within a muscle has been explored in various studies. Early histochemical studies revealed no significant difference in the proportion of slow and fast muscle fibers between deep and superficial regions of human upper and lower limb muscles [3,4]. However, others reported that fiber type distribution was not random [5],for example, with a predominance of Type II fibers at the muscle surface and Type I fibers in the deeper regions [6,7,8].

The electrical manifestation of a muscle contraction can be measured by electromyography (EMG), which has an amplitude related, in part, to the location of the motor units relative to the recording electrodes, i.e., the spatial distribution of the motor units. Beck et al. [9] observed changes in the vastus medialis EMG–force relation at various distances from the innervation zone. Trent et al. [10] also found that the shape of the biceps brachii EMG–force relation was altered as a function of the distance between the innervation zone and the recording position. The spatial sensitivity of the EMG–force relation relative to the innervation zone was also confirmed in a linear electrode array study of paretic and contralateral muscles following stroke [11]. Given that the same force signal was used for different channels, the spatial variation in EMG–force relation reflected mainly changes in EMG properties because of changes in the relative position between active motor units and the recording electrodes. While most previous simulation studies have investigated various motor unit factors that may impact the EMG–force relation [12,13,14,15], few have examined the effect of motor unit distribution within a muscle. Compared with the often-used random distribution of different motor units or muscle fiber types, Robertson and Johnston applied a weighting term to produce different fiber type content in deep and superficial muscle regions, resulting in a larger variety of EMG–force relations [16].

In this study, quantitative analyses of both simulated and experimental EMG–force relations were performed. First, we hypothesize that the EMG–force relation is sensitive to different motor unit spatial distributions. To test this hypothesis, we implemented a classic motor neuron pool model to simulate the EMG–force relation. Three different motor unit distributions with respect to muscle depth were simulated (superficial, random, and deep). For each condition, a log-transformed EMG–force relation was constructed, which has been proved useful in characterizing EMG or mechanomyographic outputs with respect to the muscle contraction level [10,17,18,19]. Second, we hypothesize that a muscle’s EMG–force relation is sensitive to different electrode positions. To test this hypothesis, we recorded high-density surface EMG signals using electrode arrays at different voluntary isometric contraction levels of the biceps brachii in healthy subjects and examined the log-transformed EMG–force relation across the electrode array recordings. The findings confirmed both hypotheses, i.e., the slope of the log-transformed EMG–force relation was found to be sensitive to different motor unit spatial distributions in the simulation study and to different proximal–distal electrode positions in the experimental study. Therefore, it may provide a convenient and useful adjunct parameter for assessing muscle or motor unit changes associated with disease, injury, or advanced aging. 

## 2. Materials and Methods

### 2.1. Simulation Study

Surface EMG and force signals were simulated using a motor unit pool model [12]. There were 150 motor units in the modeled motor neuron pool, which innervated about 70,000 muscle fibers. The number of muscle fibers per motor unit and motor unit recruitment threshold (RTE) each had an exponential distribution. Consequently, most motor units had a relatively low RTE and number of muscle fibers (range 22–2137) or twitch force (range 1–100). Once the modeled excitatory drive exceeded RTE, the motor unit started to discharge at a minimum firing rate of 8 Hz [12]. The last motor unit was simulated to be recruited at 80% or 40% maximum excitation. The former corresponded to the activity of large muscles, where recruitment plays a significant role throughout the force range (i.e., biceps brachii), whereas the latter mimicked the activity of small muscles where rate coding plays a more important role in force generation (i.e., hand muscles). The firing rate of each motor unit increased linearly with excitation until the peak firing rate (PFR) was reached. Both “onion skin” and “reverse onion skin” firing strategies were used when simulating motor unit firing behavior. The “onion skin” strategy indicates that early recruited low-threshold motor units attain higher peak firing rates than later-recruited high-threshold motor units. Although the “onion skin” pattern is the most common observation during voluntary muscle contractions [20,21], other patterns of motor unit firing rate behavior have been proposed [22,23]. Some reported similar PFR in all motor units [24] or that high-threshold units reach higher PFR than low-threshold units (reverse “onion skin” pattern) [25,26]. Therefore, both strategies were implemented in our study. The PFR of the largest and smallest motor unit was set to 35 Hz and 25 Hz, respectively. The excitation level ranged from 10% to 100% of maximum excitation with 10% increments.

**Figure 1:**
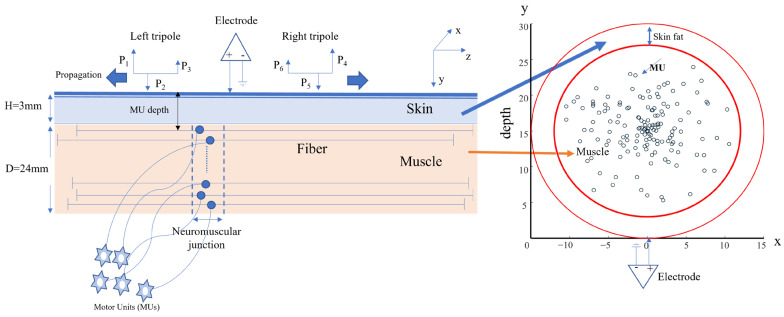
The cylindrical muscle model and electrode for surface EMG simulation. Left panel: the tripole model and the position of the electrode with respect to the cylinder parameters. Right panel: the cross-section of the cylindrical model showing muscle and skin fat layers.

A tripole model was used to simulate the generation of EMG signals [27]. In brief, the muscle was assumed to have a cylindrical shape. The *x*-axis represents the width of the muscle in the transverse direction, the *z*-axis represents the muscle fiber direction, and the *y*-axis represents the muscle depth (Figure 1). Muscle diameter was set to 24 mm, and the thickness of fat plus skin layers was 3 mm. The action potential $\varphi_{j}$, generated by fiber *j* and detected by the electrode on the surface (*x–z* plane), was given by Equation (1), where $K_{a}$, represents the ratio of electric conductivities in the *z* ($\sigma_{z}$) and *x* directions($\sigma_{x}$) (which were set to $\sigma_{z}$ = 0.33 and $\sigma_{x}$ = 0.06 S/m, respectively) [27]. A motor unit action potential (MUAP) was simulated as the sum of its constituent muscle fiber action potentials. The interspike intervals (ISI) for each motor unit were simulated as a random process with a Gaussian probability function. Surface EMG signals under different excitation levels detected by the electrode were simulated as a sparse combination of MUAP trains from all active motor units.

(1) $$\varphi_{j}=\frac{1}{2\pi \sigma_{x}}\overset{6}{\underset{i=1}{\sum }}\frac{P_{i}}{\sqrt{\left({\left(x-x_{i}\right)}^{2}+y_{i}^{2}\right)K_{a}+{\left(z-z_{i}\right)}^{2}}}$$

In this study, the electrode was simulated at the midline of the muscle (*x* = 0) (Figure 1) [27]. The sampling rate was 2000 Hz. For each motor unit (the ith motor unit), the twitch force simulation was adopted from the Fuglevand model [12], which followed a second-order critically damped impulse response (Equation (2)). The range of peak twitch force $P_{i}$ across all motor units was 100-fold, and the range of contraction time $T_{i}$ was 3-fold. Motor units with lower RTE were simulated to have lower and longer twitch forces. For example, the first recruited smallest motor unit had a *P_i_* of 1 arbitrary unit (au) and a $T_{i}$ of 90 ms, while the largest motor unit had a *P_i_* of 100 au and $T_{i}$ of 30 ms.

(2) $$f_{i,\;j}\left(t\right)=g_{i,j}*\frac{P_{i}\cdot t}{T_{i}}\cdot e^{1-\frac{t}{T_{i}}}$$

The gain $g_{i,j}$ (Equation (2)) was adjusted based on $T_{i}$ and the ISI of the discharge j. The gain was set to 1 when $T_{i}/ISI_{j}<0.4$ and otherwise was determined as in Equation (3). The total muscle contraction force was calculated as the linear summation of each motor unit force output.

(3) $$g_{i,j}=\frac{1-e^{-2{\left(T_{i}/ISI_{j}\right)}^{3}}}{T_{i}/ISI_{j}}\;\;\;\;\;\;\;\;\mathrm{when}\;\;\;T_{i}/ISI_{j}>0.4$$

**Figure 2:**
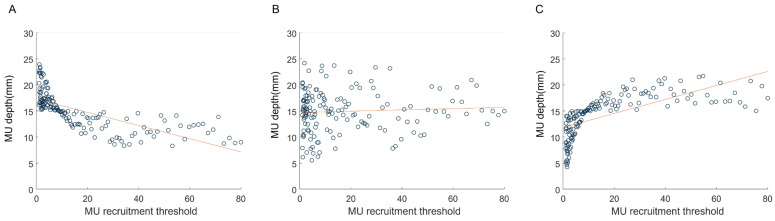
Three different motor unit distributions with respect to muscle depth. (**A**) motor unit depth is negatively related with motor unit size. (**B**) motor unit depth is randomly assigned. (**C**) motor unit depth is positively related to motor unit size.

Three different motor unit distributions, with respect to muscle depth, were simulated. The first of these were larger higher-threshold motor units, which were assumed to be preferentially located in the superficial regions of the muscle (termed “superficial”). In this case, there is a negative correlation between motor unit size and motor unit depth (Figure 2 left panel, slope of the linear regression = −0.13). The second simulation was small and large motor units, which were assumed to be randomly distributed in the muscle (termed “random”) regardless of the depth (Figure 2 middle panel, slope of the linear regression close to 0). The third simulation was larger higher-threshold motor units, which were assumed to be preferentially located in the deeper regions of the muscle (termed “deep”). In this case, there was a positive correlation between motor unit size and motor unit depth (Figure 2 right panel, slope of the linear regression = 0.12).

### 2.2. Experimental Study

#### 2.2.1. Participants and Consent

Nine subjects (8 male, 1 female, 28.9 ± 4.8 years, mean ± SD) participated in the experiment. They were recruited by word of mouth or posters and had no known history or symptoms of any neuromuscular disorder. All subjects were well informed of the experimental procedures, risks, and possible discomfort and provided written informed consent. The study was approved by the local ethics committee (ethical approval number: GWIRC-AF/SC-07/2016.20).

#### 2.2.2. Experiment Protocol

**Figure 3:**
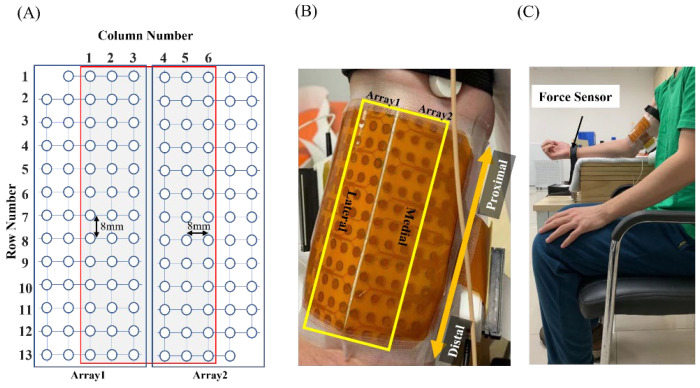
(**A**) Schematic representation of the two adhesive 2D matrices for experimental signals. (**B**) High-density electrode matrix consisting of a grid with 6 columns (that are positioned parallel to the muscle fiber direction) and 13 rows. (**C**) Illustration of the experimental setup.

Surface EMG signals were recorded from the biceps brachii muscle using two electrode arrays (Figure 3A,B). Each array (ELSCH064NM2, Bioelettronica, Torino, Italy) consists of 64 channels with an 8 mm inter-channel distance arranged in a grid of 5 columns by 13 rows (one column consisted of 12 channels and the other four of 13 channels). One array was placed over the lateral side (Matrix 1) and another over the medial side (Matrix 2) of the biceps brachii and secured with elastic straps. The columns of both arrays were placed parallel to the fiber direction. A ground channel was placed at the elbow. Subjects sat in height-adjustable chairs, each with the back fully against the backrest (Figure 3C). The dominant forearm (right side in all cases) was placed in a force-measuring device (CZL-3 T, Leitai, Bengbu, China) used to measure elbow flexion force. The elbow angle was set at 120° degrees (180° = full extension), and the forearm was supinated. The generated force was displayed on a second monitor in front of the subject as real-time feedback. Each subject performed two to three maximum voluntary contraction (MVC) trials, and the largest value of the trials was adopted as the MVC value. During the contractions, subjects were strongly encouraged to give their best effort without altering the position of the arm significantly. Each subject completed a series of 5 s submaximal contractions at 20, 40, 60, and 80% MVC, with a brief rest period (>3 min) between each to avoid muscle fatigue.

Surface EMG signals were recorded by a signal amplifier in a monopolar configuration (EMG–USB2, with a sampling frequency of 2048 Hz, 12-bit A/D converter, Bioelettronica, Torino, Italy). The EMG amplitude and mean force were calculated from a 2 s segment (4000 sample points) of each contraction. The force and EMG amplitude were normalized to their maximum levels. 

### 2.3. Log-Transformed EMG–Force Relation

When we have force and surface EMG measurement of a muscle’s isometric contraction at different levels, a linear regression model fitting can be used to describe the natural log-transformed EMG–force relation, as shown in Equation (4):

(4) $$\ln\left[Y\right]=b\left(\ln\left[X\right]\right)+\ln\left[a\right]$$

where X is the force measurement, Y is the root mean square amplitude (RMS) of surface EMG, ln represents the natural log, b is the slope, and $\ln\left[a\right]$ is the natural log of the Y intercept. The equation can also be expressed as an exponential equation after antilog transformation:

(5) $$Y=aX^{b}$$

The value of b indicates whether the original, non-transformed EMG–force relation is linear or nonlinear. If b is equal to 1, then Y increases linearly with X, if b is greater than 1, Y increases faster than X, and if b is less than 1, Y increases slower than X.

### 2.4. Data Analysis

For the simulation analysis, 50 repetitions were performed and averaged for each depth condition. A one-way ANOVA was performed to determine the effect of the depth condition on the exponent b (slope of the EMG–force relation in Equation (4)). The distribution of data was tested using the Kolmogorov–Smirnov normality test. When a significant overall effect was confirmed, the Bonferroni-corrected post-hoc test for multiple comparisons was performed.

In the experimental study, for each subject, we averaged *b* across the whole electrode array and the different areas of the array as well. The proximal area of the muscle was represented by electrodes in rows 3 and 4, and the distal area was represented by rows 10 and 11. The lateral area of the muscle was represented by electrode columns 1 and 2, and the medial area by columns 5 and 6. The Wilcoxon signed-rank test was used to test whether *b* was significantly different between the proximal and distal regions or between the lateral and medial areas. In all of the statistical analyses, a *p*-value less than 0.05 was considered to be statistically significant. The analyses were performed using MATLAB 2020b.

## 3. Results

### 3.1. Simulation of the EMG–Force Relation

Different motor unit recruitment ranges (40% and 80%of maximum excitation) and firing strategies (“onion skin” and reverse “onion skin”) were used in the motor neuron pool model. Although variation in these factors influenced the simulated EMG–force relation, as demonstrated in previous simulation studies [12,13,14,15], our data confirmed a similar trend of the slope changes of the log-transformed EMG–force relation with respect to different motor unit depth distributions (using the simulated two motor unit recruitment ranges and two firing strategies). For the sake of simplicity, the results from only one motor unit recruitment range (40% of maximum excitation) and firing strategy (reverse “onion skin”) are presented below. 

**Figure 4:**
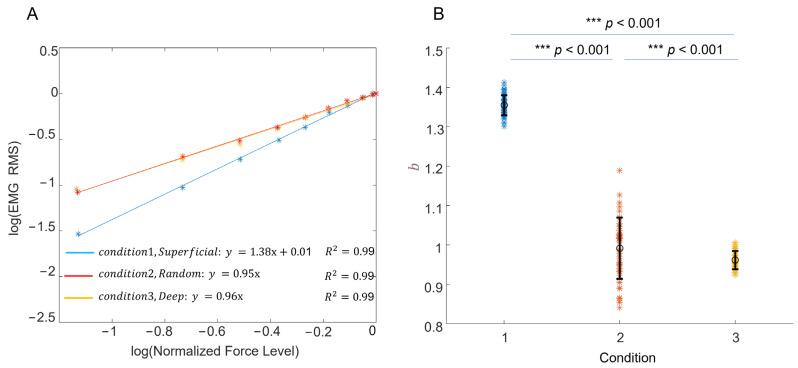
(**A**) Representative examples of log transformed EMG RMS–force relation when large motor units were assumed to preferentially located in the superficial or deep regions of the muscle or unrelated to muscle depth (random). (**B**) The results for *b* of all the repetitions in each simulated condition.

Representative examples of the log-transformed EMG–force relation under the three simulated motor unit depth distributions are shown in Figure 4. Motor unit depth distribution was found to have a significant main effect on *b* (*p* < 0.001); *b* was largest when larger motor units were located in the more superficial regions of the muscle (and the smaller units were located in the deeper regions). Post-hoc comparisons indicate that *b* for large motor units preferentially located superficially (1.38 ± 0.03, mean ± SD) was significantly higher than for the random depth (1.00 ± 0.07, *p* < 0.001) or deep depth (0.94 ± 0.02, (*p* < 0.001) conditions. The *b* for the random depth condition was also significantly larger than the deep depth condition (*p* < 0.001). 

### 3.2. Experimental Recording of the EMG–Force Relation

**Figure 5:**
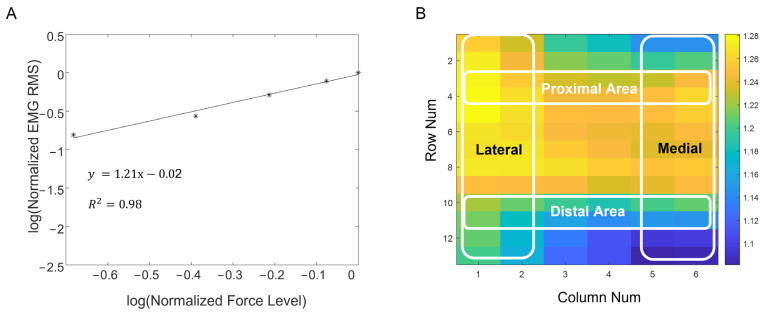
(**A**) The log transformed normalized EMG RMS–force relation (averaged from all electrodes) in a representative subject. (**B**) The distribution of *b* across all electrodes for the subject.

**Figure 6:**
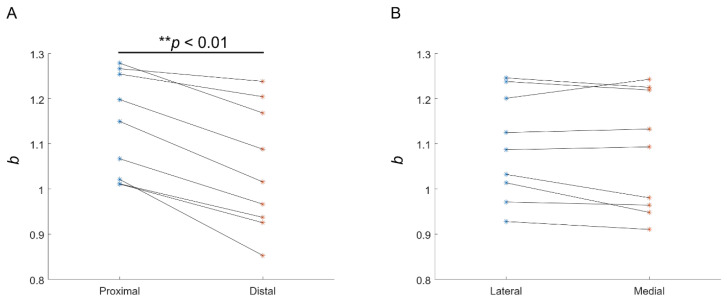
(**A**) Individual *b* for all subjects recorded from the proximal (average of all channels in rows 3 and 4) and distal (rows 10 and 11) muscle regions. (**B**) Individual *b* for all subjects recorded from the lateral (average of all channels in columns 1 and 2) and medial (columns 5 and 6) muscle regions.

The log-transformed normalized EMG–force relation from one representative subject is presented in Figure 5. EMG signals were averaged from all electrodes to represent overall muscle activity; b was 1.21, indicating that EMG amplitude increased relatively more than force (Figure 5A). The distribution of b across all channels is shown in Figure 5B for this subject. In this instance, b in the proximal region tended to be larger than in the distal region, whereas *b* was similar in the lateral and medial regions. In all subjects, *b* was larger in the proximal than distal region; mean *b* was larger proximally than distally 1.14 ± 0.11 and 1.04 ± 0.14, Z = 2.67, *p* < 0.01, Figure 6A). The effect size was 0.89, calculated as Z statistic divided by the square root of the sample size. No significant difference in b was found between the lateral (1.08 ± 0.13) and medial (1.09 ± 0.12) regions (Figure 6B).

## 4. Discussion

This study examined the EMG–force relation with respect to the depth distributions of different motor units in a muscle. The relation between EMG amplitude and muscle force has been extensively studied by others [12,13,14,15,16]. Two typical forms of the EMG–force relation have been reported [28], namely an approximately linear relation and a curvilinear relation, in which EMG amplitude increases relatively faster than force. In this study, the log transformation was used to process the EMG–force relation. The resultant slope (*b* term) can be used to characterize both linear and nonlinear forms of the relationship (or linear, logarithmic, and exponential forms [16]). Log-transformed processing has been used in previous studies when examining EMG–force relations or the mechanomyographic amplitude versus force relations [17,18,19].

We found that simulated *b* of the transformed EMG–force relation was sensitive to different motor unit depth distributions. The predominance of large motor units in the more superficial regions of the muscle tended to increase the slope. The muscle EMG–force relation reflected progressive changes in the recruitment and firing rates of the constituent motor units. The variation in *b,* due to different motor unit depth distributions, arose from EMG changes rather than changes in force. During a muscle contraction, small motor units were first recruited at relatively low forces, and large motor units were progressively activated as the force was increased. The volume conductor effect of skin and subcutaneous tissues impacted upon EMG amplitude and *b* may have been influenced by motor unit depth distribution, i.e., greater *b* in muscles with relatively larger superficial motor units. Therefore, EMG amplitude can be relatively small during low forces because less of the small (deep) motor unit potentials can be volume conducted to the muscle surface. As force is progressively increased, EMG amplitude can increase relatively more than force because more of the large (superficial) motor unit potentials can be volume conducted to the muscle surface. 

The spatial distribution of muscle fiber types has been studied previously, and the findings are mixed. Some reported that Type I and Type II fibers were randomly distributed in the muscle cross-section [3,4]. Others found a larger proportion of Type II fibers at the muscle surface [6,7,8]. In the biceps brachii, Elder and colleagues reported a higher percentage of Type II fibers in the superficial than in the deep region in all four people examined at an autopsy (group means, 58.1% vs. 52.3%), but the difference was not significant based on an ANOVA analysis [5]. However, the difference was significant when based on a paired t-test (*p* = 0.01). Similarly, in another study of six people at an autopsy, the mean biceps brachii Type II fiber percentage was higher in the superficial than in the deep region (57.7% vs. 49.5%), but statistics were not reported for these means [6]. In macro-EMG(which captures the activity of a complete motor unit) analysis at different depths of the human vastus lateralis muscle, large motor units were found to be more superficially distributed, indicative of an inverse relationship between recording depth and macro-EMG amplitude or area [29,30]. In contrast, there are also EMG studies suggesting that large motor units are located deeper in a muscle [31,32,33]. 

The high-density surface EMG used in this study provided an opportunity to observe the slope distribution of the log-transformed EMG–force relation across the electrode array. Our experimental results indicate that *b* was larger proximally than distally, whereas it was not different between the medial and lateral areas of the muscle. This is consistent with a previous linear electrode array surface EMG examination of leg extensors, where *b* was significantly higher near innervation zone channels than distal channels [10]. Although a depth gradient with motor unit size has been suggested, few studies have examined how such a gradient might change in different muscle regions. Based on our simulation results, the relatively high *b* of the log-transformed EMG–force relation observed from the proximal biceps seems to suggest a difference in motor unit spatial distribution between proximal and distal regions. From the experimental results, we might infer that muscle fibers may not be parallel to the skin over the whole muscle (fiber) length; however, they may run at an angle. Thus, fast-twitch fibers might be located more superficially in the proximal region and extend deeper into the distal portion of the muscle. Alternatively, the apparent proximal–distal dependency of *b* may simply reflect gross anatomy and the location of proximal–distal muscle regions relative to the recording electrodes.

Experimental studies of the EMG–force relation may have value in understanding motor unit plasticity associated with aging, disease, and injury. For example, changes in EMG–force relation have been reported in stroke [34,35,36,37], amyotrophic lateral sclerosis [38], and spinal cord injury [39] patients. A number of motor unit factors may collectively influence the EMG–force relation, including loss of functional motor units, impaired motor unit control properties, and fiber atrophy. Examination of the slope change of the log-transformed EMG–force relation may help understand the selective loss of motor units or changes in muscle fibers that may affect motor unit distribution [40].

Finally, yet importantly, the limitations of the study should be acknowledged. In our simulation, many simplified assumptions were used. The primary focus of the model was the spatial distribution of small and large motor units. Although a significant difference in the slope of the log-transformed EMG–relation was confirmed in the simulation by solely varying motor unit spatial distribution, there are many factors that can influence the EMG–force relation (the *b* values). For example, variations in muscle fiber length, conduction velocity, arm geometry, surface electrode size, and configuration may all have an effect on recorded surface EMG signals [41,42,43]. These physiological and recording factors should be considered for data analysis and interpretation. Furthermore, surface EMG has an intrinsic limitation in that the electrode can only record a finite portion of the muscle, notably less in larger muscles. Therefore, the experimental EMG recorded here only reflects activity in a portion of the muscle and is not necessarily applicable to the whole muscle. 

## 5. Conclusions

This study examined the spatial dependence of EMG–force relations using both simulated and experimental approaches. In the simulation study, it was found that the slope (*b*) of the log-transformed EMG–force relation was sensitive to three different motor unit depth distributions. In the experimental study, the slope distribution of the log-transformed EMG–force relation in the biceps brachii muscle showed a spatial dependence along the proximal–distal direction. The findings of this study indicate that the slope of the log-transformed EMG–force relation can provide a convenient and useful adjunct parameter in the investigation of muscle or motor unit changes associated with pathology, injury, and aging.

## Data Availability

The data that support the findings of this study are available from the corresponding author upon reasonable request.

## Abbreviations

au	arbitrary unit
EMG	electromyography
ISI	inter-spike intervals
MUAP	motor unit action potential
PFR	peak firing rate
RMS	root mean square amplitude
RTE	recruitment threshold
